# A mathematical model of parathyroid gland biology

**DOI:** 10.14814/phy2.14045

**Published:** 2019-03-29

**Authors:** Gudrun Schappacher‐Tilp, Alhaji Cherif, Doris H. Fuertinger, David Bushinsky, Peter Kotanko

**Affiliations:** ^1^ Department of Mathematics and Scientific Computing University of Graz Graz Austria; ^2^ Renal Research Institute New York City New York; ^3^ School of Mathematical and Statistical Sciences Arizona State University Tempe Arizona; ^4^ Global Research and Development Fresenius Medical Care Germany Bad Homburg Germany; ^5^ Division of Nephrology Department of Medicine University of Rochester School of Medicine Rochester New York; ^6^ Icahn School of Medicine at Mount Sinai New York City New York

**Keywords:** Mathematical model, Parathyroid hormone, Calcium‐sensing receptor, Parathyroid gland

## Abstract

Altered parathyroid gland biology in patients with chronic kidney disease (CKD) is a major contributor to chronic kidney disease‐mineral bone disorder (CKD‐MBD). This disorder is associated with an increased risk of bone disorders, vascular calcification, and cardiovascular events. Parathyroid hormone (PTH) secretion is primarily regulated by the ionized calcium concentration as well as the phosphate concentration in the extracellular fluid and vitamin D. The metabolic disturbances in patients with CKD lead to alterations in the parathyroid gland biology. A hallmark of CKD is secondary hyperparathyroidism, characterized by an increased production and release of PTH, reduced expression of calcium‐sensing and vitamin D receptors on the surface of parathyroid cells, and hyperplasia and hypertrophy of these cells. These alterations happen on different timescales and influence each other, thereby triggering a cascade of negative and positive feedback loops in a highly complex manner. Due to this complexity, mathematical models are a useful tool to break down the patterns of the multidimensional cascade of processes enabling the detailed study of subsystems. Here, we introduce a comprehensive mathematical model that includes the major adaptive mechanisms governing the production, secretion, and degradation of PTH in patients with CKD on hemodialysis. Combined with models for medications targeting the parathyroid gland, it provides a ready‐to‐use tool to explore treatment strategies. While the model is of particular interest for use in hemodialysis patients with secondary hyperparathyroidism, it has the potential to be applicable to other clinical scenarios such as primary hyperparathyroidism or hypo‐ and hypercalcemia.

## Introduction

The concentration of extracellular ionized calcium is maintained within a narrow physiologic range by an exquisite system of negative and positive feedback regulators involving the major organs that transport calcium and phosphate, that is, the intestine, the kidneys, bone, and the endocrine organs, most prominently the parathyroid gland (PTG). To avoid clinical symptoms, the arterial blood extracellular ionized calcium concentration (Ca^2+^) must be maintained within about ±1–2% (Brown 2007).

A key endocrine regulator for Ca^2+^ is parathyroid hormone (PTH) (Pocotte et al. 1991; Nemeth 2002; Silver and Naveh‐Many 2009), which increases serum calcium levels by enhancing renal tubular calcium reabsorption, stimulating net bone resorption, and increasing the production of activated vitamin D (1,25(OH)_2_D_3_) which increases net intestinal calcium absorption. PTH is produced, stored, and eventually released by the PTG. The PTG consists of two cell populations: active secretory cells and quiescent cells, which are able to proliferate or undergo apoptosis. Secretory active cells produce, store, and release PTH. PTG activity itself is mainly regulated by extracellular Ca^2+^ which activates the calcium‐sensing receptors (CaSR) on the surface of PTG cells (Pocotte et al. 1991; Schmitt et al. 1996).

The CaSR is integrally involved in PTG function and biology and, therefore, the key to understanding pathologies such as secondary hyperparathyroidism. The activation of the CaSR by the binding of Ca^2+^ initiates several signaling pathways which downregulate PTH release, production, and PTG cellular proliferation rates (Chen and Goodman 2004; Chakravarti et al. 2012; Conigrave and Ward 2013). Moreover, the CaSR regulates PTH mRNA stability (Moallem et al. 1998), thereby mediating its rate of degradation. Besides controlling PTH secretion, synthesis, degradation, and PTG proliferation, the CaSR upregulates vitamin D receptor (VDR) expression (Canalejo et al. 2000).

**Figure 1:**
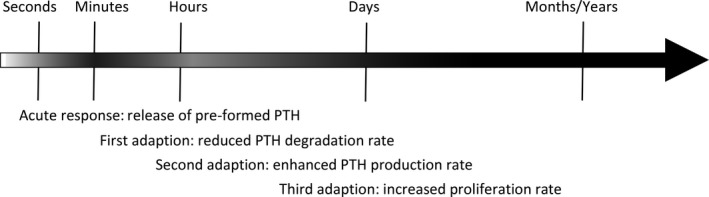
Schematic timeline for PTH secretory rate summarizing results presented in (Habener 1981; Brown et al. 1985; Schwarz et al. 1993; Tokumoto et al. 2005; Goodman and Quarles 2008). Darker color indicates higher PTH secretory rate. The adaptive mechanisms of the parathyroid gland operate on different time scales ensuring elevated PTH secretion over a long period of time.

The PTG adapts swiftly to conditions requiring enhanced PTH secretion, such as hypocalcemia. The different adaptive responses manifest on significantly different timescales, reaction times ranging from minutes to hours, days, and weeks. For instance, in the case of an acute hypocalcemia, the PTG quickly responds by releasing stored PTH within seconds to minutes (Felsenfeld et al. 2007). If hypocalcemia persists, intracellular PTH degradation rate declines within 20 min, thereby increasing the amount of intact PTH that can be released (Schwarz et al. 1993). If normocalcemia is still not attained, the PTH production rate increases within an hour (Habener 1981). Subsequently, the PTG will augment its cellular proliferation rate within 2 days (Tokumoto et al. 2005). In chronic hypocalcemia, the enhanced proliferation rate will lead to hyperplasia whereby the PTG mass increases 10–100 fold or more (Fukagawa et al. 2006) (Fig.**** 1).

In patients with chronic kidney disease (CKD), the loss of the regulatory kidney function triggers a cascade of processes eventually leading to secondary hyperparathyroidism, an abnormality characterized by increased PTH synthesis and secretion, and PTG cell proliferation. One hallmark of CKD is the impaired renal synthesis of the principal bioactive form of vitamin D, 1,25‐dihydroxyvitamin D_3_ (1,25D) and the significantly impaired renal clearance of phosphate (Levin et al. 2007). Both 1,25D and phosphate significantly influence CaSR regulation and thereby PTH synthesis and release and PTG proliferation. There is a positive feedback loop between the CaSR and the vitamin D receptor (VDR) expression (Chakravarti et al. 2012) and suppression of CaSR expression at high phosphate levels (Brown et al. 1999). The pathological effects exerted by high phosphate levels are significant. Studies in uremic rats have shown that a high phosphate diet enhances PTG proliferation within a few days (Navehmany et al. 1995; Denda et al. 1996; Slatopolsky et al. 1999). Low calcium intake has a similar effect, but the required time for PTG proliferation is significantly longer. Importantly, the positive feedback loop of CaSR and VDR is effectively diminished in the presence of high phosphate levels (Canalejo et al. 2000).

In CKD, PTH stimulates bone resorption over bone formation, resulting in a net release of Ca^2+^ and phosphate from the bone into the systemic circulation (Graciolli et al. 2017). As a consequence, PTH and phosphate levels increase even further, and expression of CaSR and VDR is depressed, thus lowering the sensitivity of the PTG to Ca^2+^ and 1,25D (Borrego et al. 1997; Gogusev et al. 1997). The altered biology eventually results in PTG hyperplasia and secondary hyperparathyroidism (Tokumoto et al. 2005; Goodman and Quarles 2008). Due to the complexity of the adaptation of PTG biology in patients with CKD on hemodialysis, a mathematical model of the PTG provides a useful tool to study secondary hyperparathyroidism and optimize treatment strategies. In tandem with mathematical models of bone turnover, intestinal calcium and phosphate absorption (Kroll 2000; Lemaire et al. 2004; Peterson and Riggs 2010), the PTG model provides a key element of an *in silico* model of secondary hyperparathyroidism in CKD and hemodialysis patients, enabling predictions about the development of chronic kidney disease‐mineral and bone disorder (CKD‐MBD) (Moe et al. 2006; Palmer et al. 2011).

The aim of this study was to develop a mathematical model comprising the main aspects of PTG biology. There are various published models of PTH (Momsen and Schwarz 1997; Raposo et al. 2002; Shrestha et al. 2010; Granjon et al. 2017), some including the adaptation mechanism of the PTG in patients with CKD (Riggs et al. 2012). However, we are not aware of a PTG model that separately captures the structure of the key adaptation mechanisms of the complex network regulating PTH in patients with CKD. We propose a model based on CaSR expression and activity regulated by Ca^2+^, phosphate, and 1,25D. Our model includes the various mechanisms ensuring enhanced PTH levels acting on different timescales, thereby allowing predictions for both rapid responses, for example, in the case of induced acute hypocalcemia and long‐term adaptations reflecting the transition of the normal PTG into a hyperplastic gland with reduced sensitivity to Ca^2+^ and 1,25D. We validated the model predictions with published data.

## Mathematical Model

### Key features

The core of our model is the CaSR. We make use of physiological principles governing the signaling cascade triggered by the CaSR: (1) If all key parameters (i.e., Ca^2+^, 1,25D, and phosphate) are within their optimal physiologic range, the CaSR signaling will ensure that PTH release rate, production rate, and proliferation rate are downregulated to their basal values and intracellular degradation rate is constant. (2) If one or more key parameters are not in their optimal range for a critical amount of time, CaSR signaling is altered resulting in PTG adaptations regarding PTH release rate, intracellular PTH degradation rate, PTH production rate, and cellular proliferation. The critical amount of time is significantly different for the different CaSR signaling pathways and is seconds for the release rate, minutes for the degradation rate, hours for the production rate, and days for the proliferation rate. (3) Due to the feedback loops acting on the CaSR, the alteration in signaling also changes CaSR expression over time. Less CaSR expression leads to a lower sensitivity of the PTG to blood ionized calcium concentration. (4) We use stimulus functions describing the deviation from the optimal range. Negative stimulus corresponds to values below the optimal range; positive stimulus corresponds to values above the optimal range. (5) Stimulus functions are the same for all adaptation mechanisms. Stimulus functions are chosen such that small deviations from the optimal value will not trigger a response unless these deviations last for an extended period of time. (6) All effects but hyperplasia are reversible. If a condition like acute hypocalcemia is resolved, PTH synthesis rate, intracellular degradation rate, and cellular proliferation rate will return to baseline (Brown 2007; Vervloet et al. 2017). However, since apoptosis rate is assumed to be constant (Navehmany et al. 1995; Takahashi et al. 2002), the PTG size will not decrease.

### Input parameters

**Figure 2:**
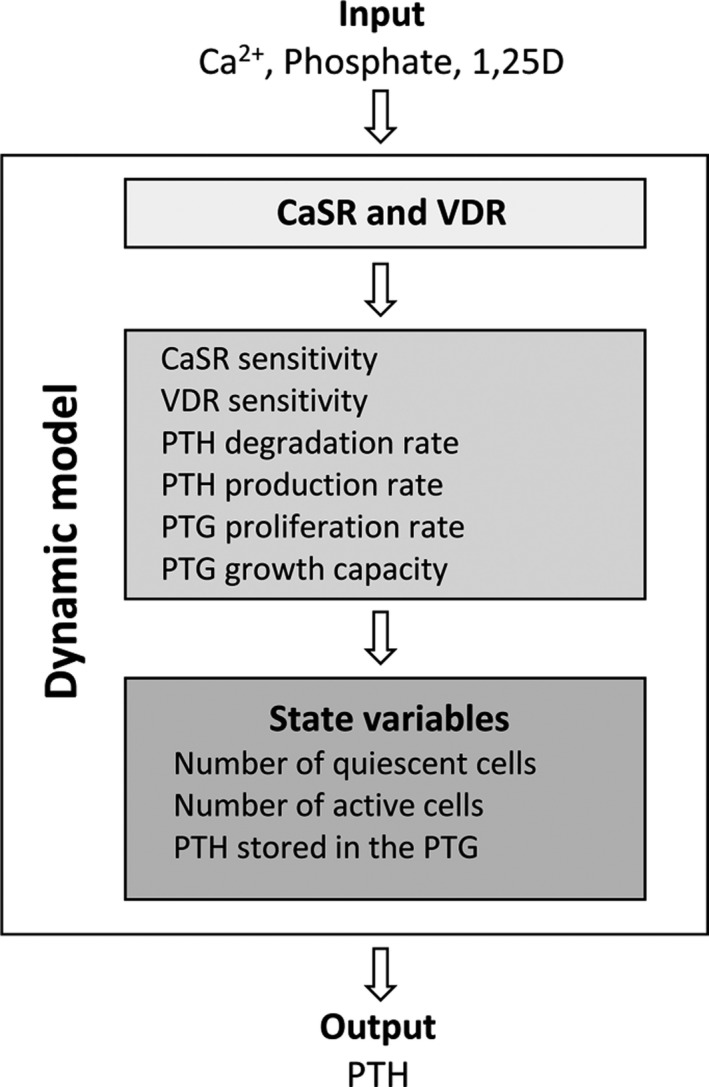
The model inputs are the plasma ionized calcium concentration, plasma phosphate concentration as well as plasma 1,25D concentration. The model output is the PTH concentration.

Ionized calcium concentration (Ca^2+^), phosphate (P), and 1,25D are the important input parameters. All Ca^2+^, 1,25D, and P profiles used in PTH predictions are simulated profiles within physiological meaningful ranges. A high‐level sketch of the model is provided in Figure 2 **.**

### Ca^2+^

The ionized calcium concentration (provided as an input parameter in this manuscript) regulates the PTG response via the CaSR. We couple the actual ionized calcium concentration and the PTG via the CaSR by equations (1), (2), (3), (4), (5), (6), (7), (8).

### CaSR expression

**Figure 3:**
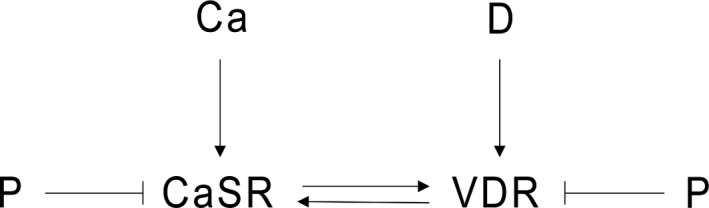
Expression of the CaSR and VDR. While there is a positive feedback loop between the VDR and CaSR, CaSR and VDR expressions are suppressed by phosphate.

CaSR and VDR expressions are determined in order to assess the overall sensitivity of the receptors to the concentration of the arterial blood ionized calcium and 1,25D concentrations. We model a dimensionless CaSR and VDR expression. The optimal value is normalized to 1 for both variables. Values lower than 1 correspond to a loss of CaSR or VDR expression. Receptor expression is regulated by a positive feedback loop between the CaSR and the VDR (Fig. 3) and is suppressed by phosphate. We therefore assume that the dimensionless CaSR expression, CaSR, and VDR expression, VDR are governed by the following system of differential equations:

(1) $$\begin{array}{c} \frac{\mathrm{dCaSR}}{dt=p_{\mathrm{Ca}}\cdot \left(\chi^{\mathrm{in}}+\left(\mathrm{VDR}-1\right)-\pi^{\mathrm{in}}\right)\cdot \mathrm{CaSR}+n_{\mathrm{Ca}}\cdot \left(1-\mathrm{CaSR}\right)} \end{array},$$

(2) $$\begin{array}{cc} \frac{dVDR}{dt}= & p_{D}\cdot \left(\delta^{\mathrm{in}}+\left(\text{CaSR}-1\right)-\pi^{\mathrm{in}}\right)\cdot \mathrm{VDR}+n_{D}\cdot \\ & (1-\mathrm{VDR}), \end{array}$$

(3) $$\frac{d\chi^{\mathrm{in}}}{dt}=\left({\text{stim}}_{C}\left(CS-Ca_{\mathrm{opt}}\right)\cdot \left(1-\mathrm{sign}\left({\text{stim}}_{C}\left(CS-Ca_{\mathrm{opt}}\right)\right)\chi^{\mathrm{in}}\right)-\chi^{\mathrm{in}}\right)\cdot \tau_{C},$$

(4) $$\begin{array}{cc} \frac{d\delta^{\mathrm{in}}}{dt}=\; & ({\text{stim}}_{D}\left(DS-D_{\mathrm{opt}}\right)\\ & \cdot \left(1-\mathrm{sign}(\left({\text{stim}}_{D}\left(DS-D_{\mathrm{opt}}\right)\right)\delta^{\mathrm{in}})\right)-\delta^{\mathrm{in}})\cdot \tau_{D} \end{array},$$

(5) $$\begin{array}{cc} \frac{d\pi^{\mathrm{in}}}{dt}=\; & ({\text{stim}}_{P}\left(P-P_{\mathrm{opt}}\right)\\ & \cdot \left(1-\mathrm{sign}\left({\text{stim}}_{P}\left(P-P_{\mathrm{opt}}\right)\right)\pi^{\mathrm{in}}\right)-\pi^{\mathrm{in}})\cdot \tau_{P} \end{array},$$

where $Ca_{\mathrm{opt}}$ $\;D_{\mathrm{opt}}$ and $P_{\mathrm{opt}}$ are the optimal blood values of Ca^2+^, 1,25D, and phosphate. CS and DS are defined in equations (6) and (8) and couple the actual blood values of Ca^2+^ and 1,25D with the receptor density. P is the serum phosphate concentration. The intensity of the stimulus and feedback is governed by *p*_Ca_ and *p*_D,_ the relationship between *p*_Ca_ and *n*_Ca_ as well as *p*_D_ and *n*_D_ determines the equilibrium of the system. $\chi^{\mathrm{in}}$ $\delta^{\mathrm{in}}$ and $\pi^{\mathrm{in}}$ are factors determining the effect of the stimulus of calcium, 1,25D, and phosphate on the CaSR and VDR respectively. The time constants$\tau_{c}$ $\tau_{D}$ and $\tau_{P}$ determine the convergence rate to the steady state after a stepwise change in ionized calcium, 1,25D, or phosphate. They are associated with the critical time after which the system starts to react to nonoptimal concentrations of ionized calcium, 1,25D, or phosphate.

We use a stimulus function which introduces autoregulation regarding long‐term effects.

The stimulus functions can be written as

$${\text{stim}}_{w}\left(x\right)=\frac{1}{1+\exp(-K_{w}\left(x-W_{1}\right))}+\frac{1}{1+\exp(-K_{w}\left(x+W_{1}\right))}-1,\text{and}W\in \left(C,D,P\right),$$

where K and W_1_ are constants. Under optimal conditions, the stimulus functions are zero for ionized calcium, 1,25D, and phosphate. Therefore, *χ* ^*in*^, *δ* ^*in*^, and *π* ^*in*^ are zero and the CaSR and VDR expressions are not altered. If the deviation from the optimal range is shorter than the critical time associated with the time constants, for example, a brief induced hypocalcemia, the stimulus on $\chi^{\mathrm{in}}\;$is too small to change $\chi^{\mathrm{in}}\;$ significantly. Therefore, CaSR expression will not be altered. However, in the case of a mild but chronic hypocalcemia, CaSR expression will slowly decline after a time lag due to the small but constant stimulus of *χ* ^in^. The terraced form of the stimulus function with the small plateau around the optimal values ensures that an insignificant deviation from the optimal values does not trigger the full response of the system such as the increase of cellular proliferation rate. Therefore, the whole system is stabilized but still able to react properly if the deviation is increasing or persistent over a long period of time. The stimulus function coupled with the delayed response is a simplification of the three‐state dimer receptor model, for example (Brea et al. 2009). The formulation of stimulus functions and delayed response replaces well‐established but numerically highly unstable delay equations.

### Effect of CaSR and VDR density

A lower density of CaSR leads to less downstream signaling. Therefore, the same actual Ca^2+^ concentration can lead to different PTG responses in a normal, healthy PTG compared to a gland with reduced CaSR expression (Goodman et al. 1998; Schmitt and Schaefer 1999). The gland with reduced CaSR expression appears less sensitive to Ca^2+^. Therefore, it is not the present ionized calcium level that determines the response of the system but rather the calcium level associated with the CaSR density which is referred to as sensed calcium concentration CS. The same holds for the 1,25D concentration. A gland with reduced VDR expression appears less sensitive to 1,25D and it is not the actual 1,25D concentration but the 1,25D value associated with the VDR sensitivity (DS) which determines the response of the system.

(6) $$\frac{dCS}{dt}=\text{sens}\left(\mathrm{CaSR}+\mathrm{VDR}\right)C-CS,$$

(7) $$\frac{dDS}{dt}=\text{sens}\left(\mathrm{CaSR}+\mathrm{VDR}\right)D-DS,$$

(8) $$\text{sens}\left(x\right)=A_{S}+\left(1-A_{S}\right)\cdot \left(x/2\right),$$

where C is the blood ionized calcium concentration and D is the 1,25D plasma concentration. At optimal conditions, the dimensionless CaSR and VDR expressions equal 1, and CS and DS equilibrate to C and D Hence, the sensed calcium concentration equals the actual blood Ca^2+^ concentration. If the dimensionless CaSR expression starts to decline, the sensed calcium concentration CS declines as well but with a slight delay. The maximal rate of decline is *A*_*S*_. This effect is reversible. If the CaSR expression increases again, for example, by long‐term effects of a low phosphate diet, 1,25D supplements, or calcimimetics, the sensed calcium concentration CS will approach C again.

### CaSR signaling

CaSR signaling is directly governed by the sensed ionized calcium concentration CS and phosphate (Silver and Naveh‐Many 2009). Furthermore, the activity is indirectly governed by VDR due to the feedback loop between the VDR and CaSR:

(9) $$\frac{d\chi_{\circ }}{dt}=p_{\circ }\cdot \left(\chi_{0}^{\mathrm{in}}-\pi_{{}^{0}}^{\mathrm{in}}\right)\cdot \chi_{\circ }+n_{\circ }\cdot \left(1-\chi_{\circ }\right),$$

(10) $$\frac{d\chi_{\circ }^{\mathrm{in}}}{dt}=\left({\text{stim}}_{\mathrm{Ca}}\left(CS-Ca_{\mathrm{opt}}\right)\cdot \left(1-\frac{{\text{stim}}_{\mathrm{Ca}}}{|{\text{stim}}_{\mathrm{Ca}}|}\chi_{\circ }^{\mathrm{in}}\right)-\chi_{\circ }^{\mathrm{in}}\right)\cdot \tau_{\mathrm{Ca}}^{\circ },$$

(11) $$\frac{d\pi_{\circ }^{\mathrm{in}}}{dt}=\left({\text{stim}}_{P}\left(P-P_{\mathrm{opt}}\right)\cdot \left(1-\frac{{\text{stim}}_{P}}{|{\text{stim}}_{P}|}\pi_{\circ }^{\mathrm{in}}\right)-\pi_{\circ }^{\mathrm{in}}\right)\cdot \tau_{P}^{\circ }.$$

Again, $p_{\circ }$ and $n_{\circ }$ determine the intensity of the stimulus on the activity, whereas the time constants $\tau_{\mathrm{Ca}}^{\circ }$ and $\tau_{P}^{\circ }$ determine the critical time with which the system responses to the stimulus. We use these sets of equations for the cellular degradation rate (∘=d), PTH production rate $\left(\circ =p\right),$ and cell proliferation rate (∘=pr)

### Parathyroid hormone

**Figure 4:**
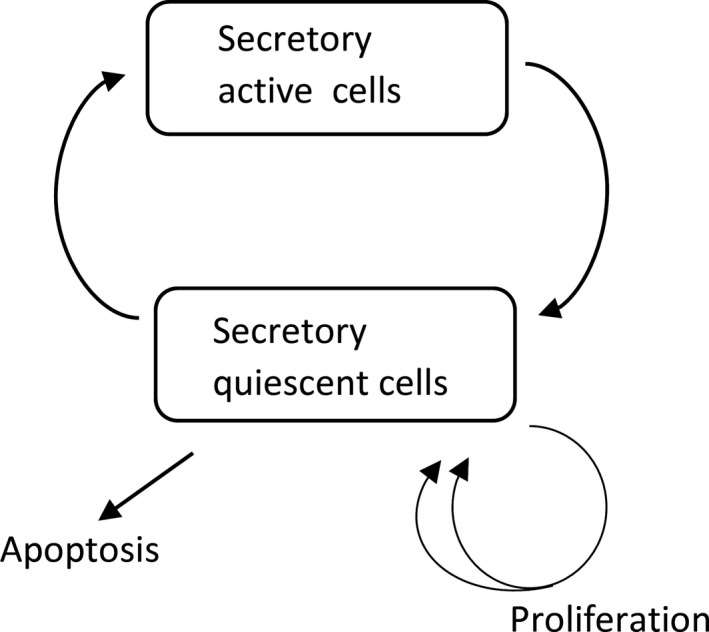
Sketch of the PTG. Cells in the secretory quiescent state can proliferate or undergo apoptosis.

There is a well‐known sigmoidal relationship between Ca^2+^ and PTH release rate (Grant et al. 1990; Felsenfeld et al. 2007). We use a previously published model for PTH allowing the storage of PTH in the gland (Brown 1983; Momsen and Schwarz 1997; Schwarz et al. 1998; Raposo et al. 2002; Shrestha et al. 2010):

(12) $$\frac{d\mathrm{PTG}}{dt}=k_{p}S-\text{release}\left(CS\right)\mathrm{PTG}-k_{d}\mathrm{PTG},$$

(13) $$\frac{d\mathrm{PTH}}{dt}=\text{release}\left(CS\right)\mathrm{PTG}-k_{\mathrm{cl}}\mathrm{PTH},$$

(14) $$\text{release}\left(CS\right)=B+\left(A-B\right)\frac{1}{1+{\left(CS/S_{r}\right)}^{m}},$$

where PTG and PTH are the PTH concentrations in the gland and serum, respectively. In healthy subjects, with phosphate and 1,25D levels in the normal range, PTG will react quickly to changes in ionized calcium concentrations by either releasing a large amount of PTH in the case of a fall in Ca^2+^ or by suppressing the release of PTH in the case of excess Ca^2+^ concentrations. However, if CaSR sensitivity is reduced, that is, in the case of chronic hyperphosphatemia or reduced 1,25D plasma levels, the release function is automatically shifted, mimicking a shift in the set‐point *S*_*r*_ (Fig. 4). The PTG cannot react properly to normal Ca^2+^ levels or even to slightly higher Ca^2+^ concentrations.

### Parathyroid gland cell populations

**Figure 5:**
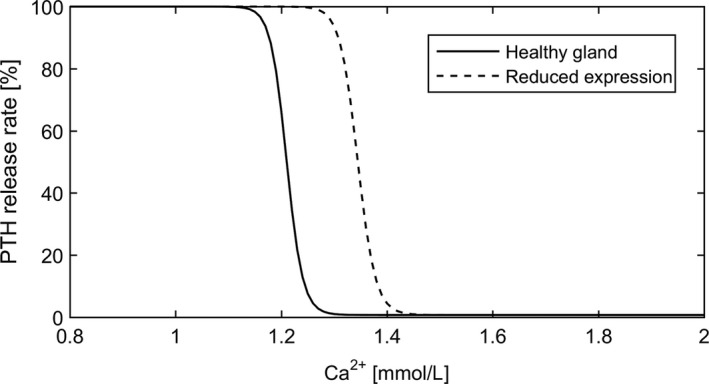
PTH release rate as a function of Ca^2+^. A reduced expression of CaSR results in a shift of the release function. In this example, *CS* equals 90% of the actual ionized calcium concentration.

Our PTG model employs two cell populations, active secretory cells S and quiescent cells Q. PTG cells cycle through the two states (Fig. 5). Cells in the quiescent state can proliferate or undergo apoptosis and – when left untreated – enhanced cell proliferation will lead to PTG hyperplasia. We capture this behavior by a growth model with dynamic carrying capacity *K* (Ribba et al. 2011; Benzekry et al. 2014). Associating the total number of cells in the quiescent state with the volume, we use the following model:

(15) $$\frac{\text{dS}}{dt}=-k_{\mathrm{SQ}}S+k_{\mathrm{QS}}Q,$$

(16) $$\frac{\text{dQ}}{dt}=k_{\mathrm{SQ}}S-k_{\mathrm{QS}}Q-k_{a}Q+k_{\mathrm{pr}}Q\ln\left(K/(S+Q)\right),$$

(17) $$\frac{dK}{dt}=k_{k}{\left(\left(S+Q\right)/\left(S_{0}+Q_{0}\right)-1\right)}^{2/3},$$

where *k*_SQ_ and *k*_QS_ are constant rates determining the cell cycle between the secretory active and quiescent state, *k*_a_ is the constant apoptosis rate, and the constant *k*_k_ is the rate with which the dynamic growth capacity *K* adjusts to the size of the gland. The size of the healthy gland is estimated by the initial steady state S_0_ = S(0), Q_0_ = Q(0).

### Intracellular degradation rate of PTH

Under normal conditions (i.e., *C*_d_ ≥ 1), CaSR keeps the cellular degradation of PTH *k*_*d*_ at a basal rate of *A*_*d*_. If not regulated by the CaSR, this rate will decrease to a minimum rate of *B*_d_ = 0.5 · *A*_*d*_ (Brown 2015):

(18) $$k_{d}=B_{d}+\left(A_{d}-B_{d}\right)\cdot C_{d}\;for\;C_{d}<1,$$

where *A*_d_ and *B*_d_ are constants.

### PTH production rate

PTH is constantly produced by secretory active PTG cells with a basal rate of *A*_p_. Again, if not downregulated by the CaSR, the production rate *k*_p_ will increase within minutes to hours to a maximum level of *B*_p_ = 2·*A*_p_ (Brown 2015):

(19) $$k_{p}=B_{p}+\left(A_{p}-B_{p}\right)\cdot C_{p}\;for\;C_{p}<1,$$

where *A*_p_ and *B*_p_ are constants.

### Proliferation rate

The cell proliferation rate *k*_pr_ increases from the basal level *A*_*pr*_ to a maximum rate of *B*_pr_ = 2 · *A*_pr_ if not downregulated by the CaSR (Wang et al. 1997; Tokumoto et al. 2005):

(20) $$k_{\mathrm{pr}}=B_{\mathrm{pr}}+\left(A_{\mathrm{pr}}-B_{\mathrm{pr}}\right)\cdot C_{\mathrm{pr}}\;for\;C_{\mathrm{pr}}<1,$$

where *A*_*pr*_ and *B*_*pr*_ are constants.

### Parameters and sensitivity analysis

The only parameters estimated for the simulations are the ones associated with the intensities of the feedback systems (Equations (1),(2),(6), and (9)).

All basic parameters for PTG, PTH release, and intracellular degradation are based on literature (Table 1). Basic parameters for the stimulus functions (Table 2) are chosen such that the derivative of the stimulus function is maximal around the critical values (Ca^2+^ (arterial blood ionized calcium)): 2.8 mg/dL, 1,25D: 90 ng/mL, P: 6.6 mg/dL) and the mean derivative in the reference range (Ca^2+^ : 4.4–5.5 mg/dL, 1,25D: 20–60 ng/dL, P: 3.4–4.5 mg/dL) is smaller than 0.08 for Ca^2+^ and P and 0.0035 for 1,25D. The value for 1,25D is much smaller since it is not as tightly regulated as the other two parameters.

**Table 1:** Basic set of parameters for the parathyroid gland, PTH release, and intracellular degradation

Compartment	Parameter	Value	Source
PTG	$k_{\mathrm{SQ}}$	2$\cdot 10^{-3}$/min	Raposo et al. (2002)
	$k_{\mathrm{QS}}$	5$\cdot 10^{-4}$ min${}^{-1}$	Raposo et al. (2002)
	$k_{p}^{0}$	3$\cdot 10^{-3}$ /min	Wang et al. (1997)
	$k_{a}$	0.001 $min^{-1}$	Wang et al. (1997)
PTH release	A	0.12 pmol/min	Shrestha et al. (2010)
	B	0.001 pmol/min	Shrestha et al. (2010)
	$S_{r}$	1.1881 mmol/L	Shrestha et al. (2010)
	m	50	(Shrestha et al. 2010)
Clearance	$k_{c}$	0.632/min	Shrestha et al. (2010)
Degradation	$A_{d}^{0}$	0.012/min	Shrestha et al. (2010)
Production	$A_{\mathrm{pr}}$	132	Shrestha et al. (2010)
Proliferation	$A_{p}$	0.003/min	Fukagawa et al. (1990)
	$k_{k}$	$5\cdot 10^{-5}$	

*k*_QS_ is chosen such that the fraction of active cells at steady state is 0.2.

**Table 2:** Basic set of parameters for the stimulation functions

Compartment	Parameter	Value
Optimal values	C	5 mg/dL
	P	4.5 mg/dL
	D	45 pg/mL
Stimulus	$C_{1}$	2 mg/dL
	$K_{C}$	2 dL/mg
	$D_{1}$	45 ng/ml
	$K_{D}$	0.2 mL/ng
	$P_{1}$	2.5 mg/dL
	$K_{P}$	2.5 dL/mg

Time constants are chosen such that the effect of the stimulus is physiologically appropriate (Table**** 3). All alterations with the exception of cellular proliferation reach a steady state. Therefore, the only time constant relevant for long‐term adaptations is the one associated with cellular proliferation. We assessed the impact of uncertainties in the time constant by multiplying or dividing the base time constants (Equations (10) and (11)) governing the response of the cellular proliferation rate to the stimulus (Equation (14)) one at a time by a factor of 10 while keeping all other parameters constant and simulating a mild hypocalcemia and hyperphosphatemia. We then analyzed the corresponding PTH predictions. The analysis revealed that the system is not sensitive to the alterations of the time constants. Even after 30 days, the difference in PTH is below 0.009% for changes in phosphate‐related values and below 0.003% for changes in calcium‐related values.

**Table 3:** Time constants and intensity parameters for stimulation functions. All parameters are unitless

Compartment	Parameter	Value
Degradation	$\tau_{\mathrm{Ca}}$	$10^{-1}$
	$\tau_{P}$	$5\cdot 10^{-2}$
	$p_{\mathrm{Ca}}$	25
	$n_{\mathrm{Ca}}$	0.5
Production	$\tau_{\mathrm{Ca}}$	$10^{-3}$
	$\tau_{P}$	$10^{-4}$
	$p_{\mathrm{Ca}}$	25
	$n_{\mathrm{Ca}}$	0.5
Proliferation	$\tau_{\mathrm{Ca}}$	$5\cdot 10^{-5}$
	$\tau_{P}$	$5\cdot 10^{-4}$
	$p_{\mathrm{Ca}}$	25
	$n_{\mathrm{Ca}}$	0.5
Expression	$\tau_{\mathrm{Ca}}$	$10^{-4}$
	$\tau_{P}$	$10^{-1}$
	$\tau_{D}$	$10^{-4}$
	$p_{\mathrm{Ca}}$	5$\cdot 10^{-3}$
	$n_{\mathrm{Ca}}$	5$\cdot 10^{-3}$
	$p_{D}$	5$\cdot 10^{-7}$
	$n_{D}$	5$\cdot 10^{-7}$

**Figure 6:**
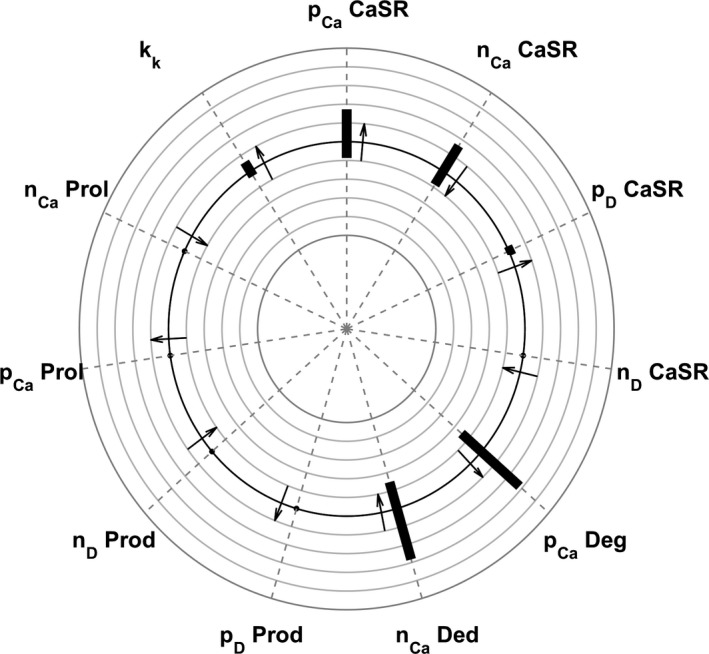
Sensitivity plot of PTH predictions to changes in various parameters. The plot depicts the maximum deviation from reference PTH values as the ratio between the calculated PTH and the reference PTH. The smallest circle corresponds to a relative deviation of 0.5, the largest circle to a relative deviation of 1.5. Arrows pointing outward indicate that higher parameter values lead to higher PTH values; arrows pointing inward indicate that higher parameter values lead to smaller PTH values.

The sensitivities of PTH predictions related to uncertainties of intensities of the stimulation functions where assessed by multiplying or dividing the base values by a factor of 2 one at a time while keeping all other parameters constant. PTH was simulated for 7 days under the condition of normocalcemia, developing hyperphosphatemia, and declining 1,25D levels. The analysis revealed the highest sensitivity to changes of *p*_*Ca*_ and *n*_*Ca*_ associated with PTH production rate (Equations (9), (13)) and to changes of *p*_*Ca*_ and *n*_*Ca*_ of the CaSR (Equations (1), (2)) determining the steady state of the CaSR expression. The sensitivity plot is presented in Figure 6.

Steady state values are directly calculated from the equations. For our basic set of parameters, these values are *S*_0_ = 0.1934, *Q*_0_ = 0.7738, *K*_0_ = 1, and *PTG*_0_ = 565. The steady state concentration of PTH is 3.15 pmol/L. Since we start at the optimal condition, all steady state values of the stimulus functions (e.g., $\chi_{\circ }^{\mathrm{in}}$) are zero and all steady state values of the functions accounting for the effect of the stimulus on the receptor (e.g., *χ*_0_) are 1.

## Results

### Acute response

The acute response of PTH to hypocalcemia depends on the rate of Ca^2+^ reduction (Brent et al. 1988; Conlin et al. 1989; Grant et al. 1990; Schmitt et al. 1996; Estepa et al. 1999; Felsenfeld et al. 2007). In a study in dogs, a linear drop by 1.6 mg/dl Ca^2+^ within 30 minutes produced a prominent PTH peak. A linear drop of the same amount within 120 minutes did not produce a prominent PTH peak but higher PTH concentrations after around 60 minutes (Estepa et al. 1999; Felsenfeld et al. 2007) Figure**** 7. The same characteristics regarding the fast decline of Ca^2+^ were found in rats (Schmitt et al. 1996). The absence of PTH overshoot in CKD patients was reported by Messa and coworkers (Messa et al. 1994).

**Figure 7:**
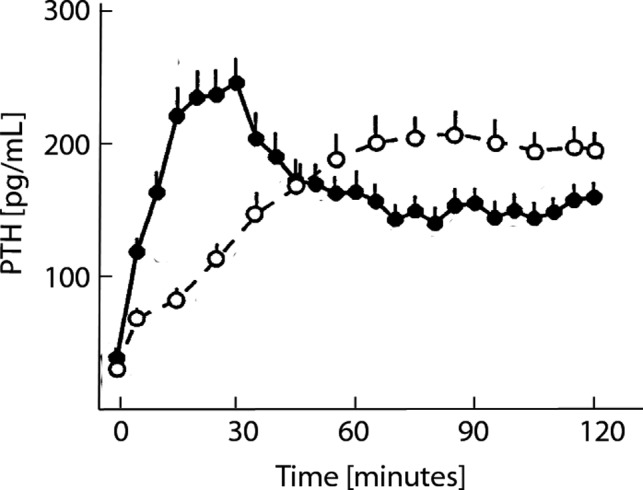
Experimental data reported in Estepa et al. (1999), Figure 2B (adjusted and reproduced with permission of Kidney International). Ionized calcium concentrations were reduced by 0.4 mmol/L either in 30 min followed by a 90‐min hypocalcemic clamp (fast reduction, solid dots) or in 120 min (slow reduction, open dots). The fast Ca^2+^ reduction leads to a prominent peak in PTH levels. However, PTH levels during the hypocalcemic clamp following the fast reduction were significantly lower than PTH levels in the slow induction group.

**Figure 8:**
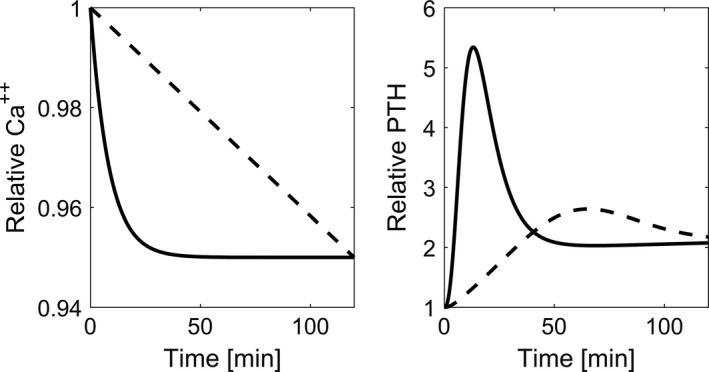
Time versus Ca^2+^ and predicted PTH concentrations during induced hypocalcemia. While PTH response shows a prominent peak when the rate of calcium reduction is high (solid line), the peak is almost diminished when the rate of change is low (dashed line).

We simulate acute Ca^2+^ change by starting from the system steady state and changing the ionized calcium concentration at different rates. Due to the storage of PTH in the secretory active cells, fast induction of hypocalcemia (5% reduction of Ca^2+^ within 30 minutes) leads to the clinically observed PTH overshoot followed by a sharp decline, while slow induction of hypocalcemia (5% reduction of Ca^2+^ within 120 minutes) leads to a small PTH overshoot before reaching levels similar to those of the fast reduction protocol (Fig.**** 8).

### Hysteresis

**Figure 9:**
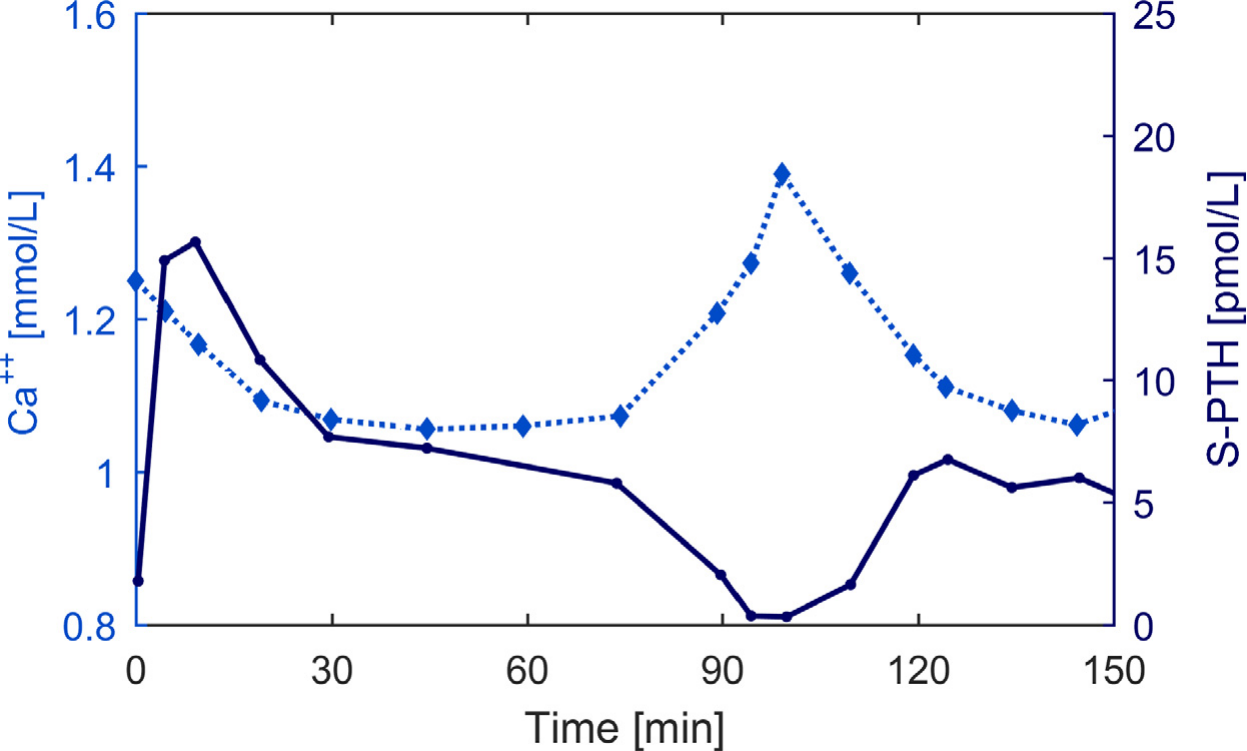
Clinical data adapted from (Schwarz et al. 1998), Figure 1(a), with permission by Clinical Endocrinology. Time versus Ca^2+^ (dotted line) and PTH (solid line) concentrations during induced hypocalcemia, normocalcemia, and subsequent hypocalcemia.

Schwarz and coworkers (Schwarz et al. 1998) used induced hypocalcemia followed by a brief return to normocalcemia and a subsequent hypocalcemia to analyze PTH hysteresis effects. After a sharp increase of PTH during the first peak of 0.8 mg/dl from Ca^2+^ baseline levels and a slight drop due to the return to normal, the authors did not observe a second transient PTH peak (Fig. 9).

**Figure 10:**
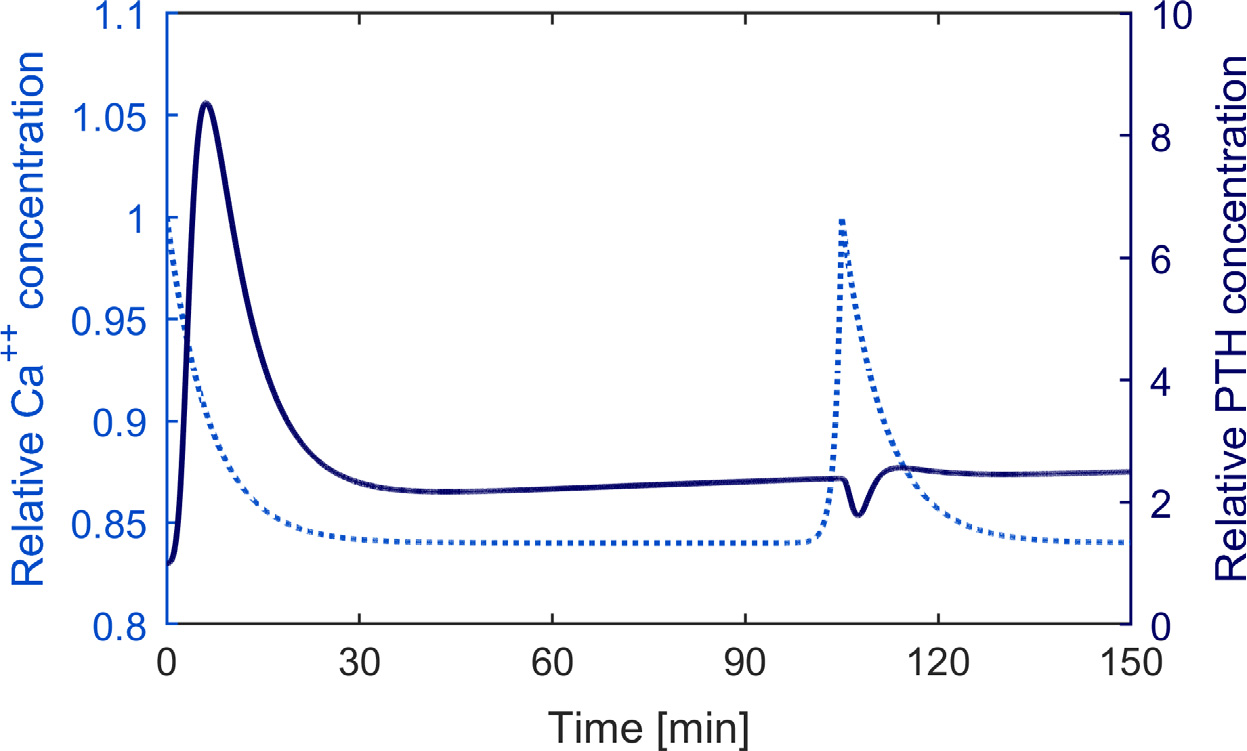
Time versus prescribed Ca^2+^ (dotted line) and predicted PTH concentrations (solid line) during simulated induced hypocalcemia, normocalcemia, and subsequent hypocalcemia.

We simulate this protocol and find the same PTH dynamics – a prominent peak during the first hypocalcemia (due to the quick release of PTH stored in the PTG cells) and no peak during the second hypocalcemia (since recovery time in the normocalcemic condition is insufficient to load the PTH storage again) (Fig.**** 10).

### Secondary hyperparathyroidism

**Figure 11:**
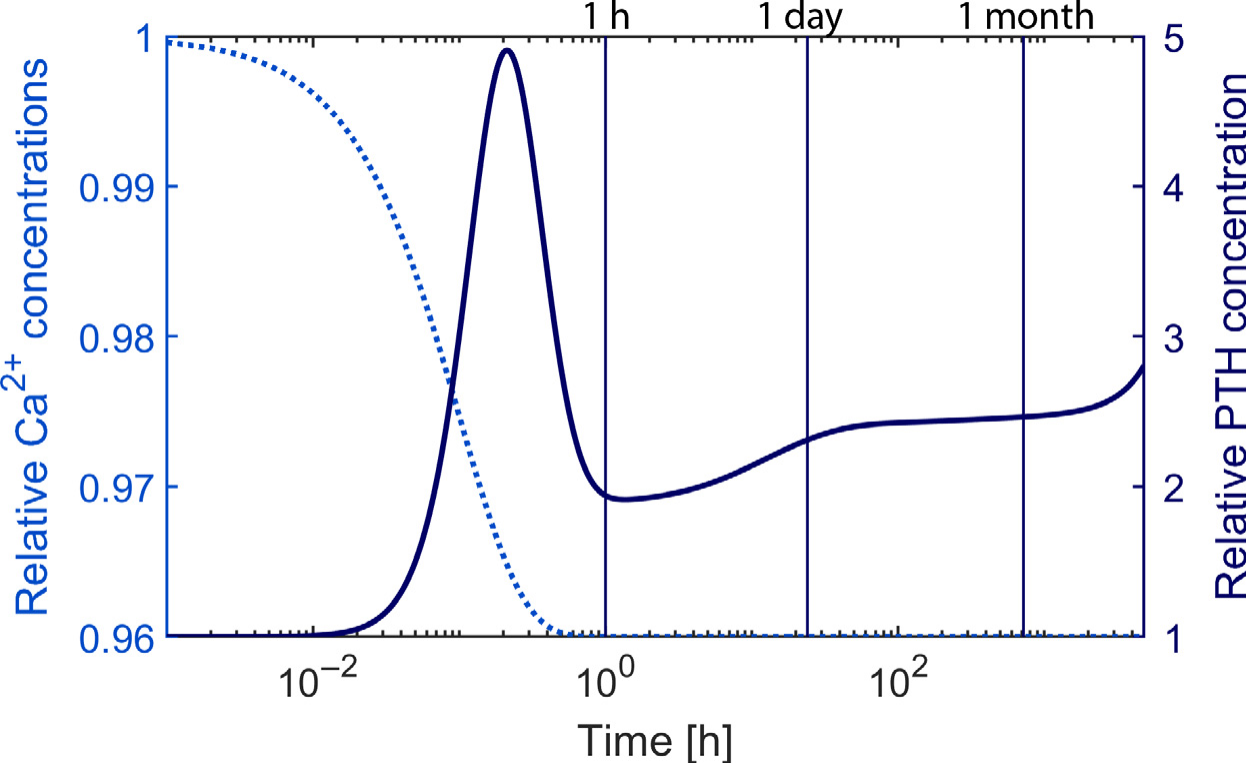
Time versus prescribed Ca^2+^ (dotted line) and predicted PTH (solid line) concentrations during simulated induced chronic hypocalcemia. The timescale is logarithmic; the black vertical lines indicate 1 h, 1 day, and 1 month.

Mild chronic hypocalcemia will trigger all mechanisms resulting in elevated PTH levels (Brown 2015) (Fig.**** 1). We simulate chronic hypocalcemia by lowering Ca^2+^ by 0.25 mg/dL and keeping it low for 200 days. Due to the slight decline in CaSR expression, the decrease in intracellular degradation, the increase in PTH production rate, and the slight increase in cellular proliferation, PTH levels will not reach a steady state but rather increase slightly but steadily (Fig.**** 11).

**Figure 12:**
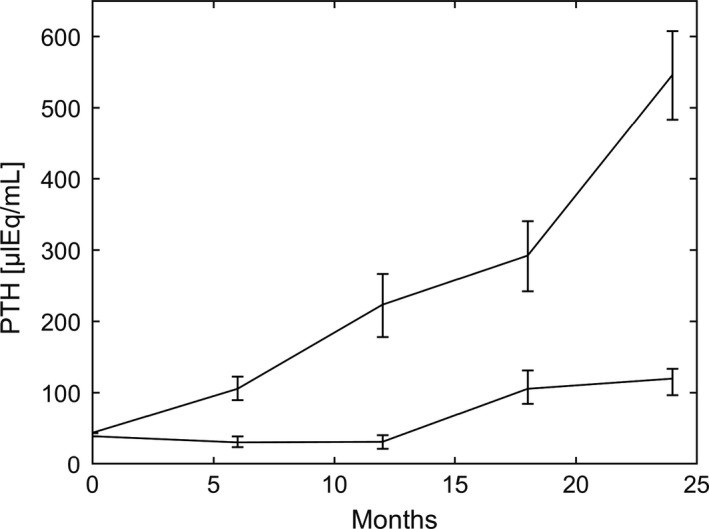
PTH (mean and range) as a function if time reported in Rutherford et al. (1977), Figure 5. Adapted and reproduced with the permission of the Journal of Clinical Investigation. PTH was reported every 6 months. The slope of increase was significantly lower if phosphate intake was reduced according to the fall in glomerular filtration rate.

**Figure 13:**
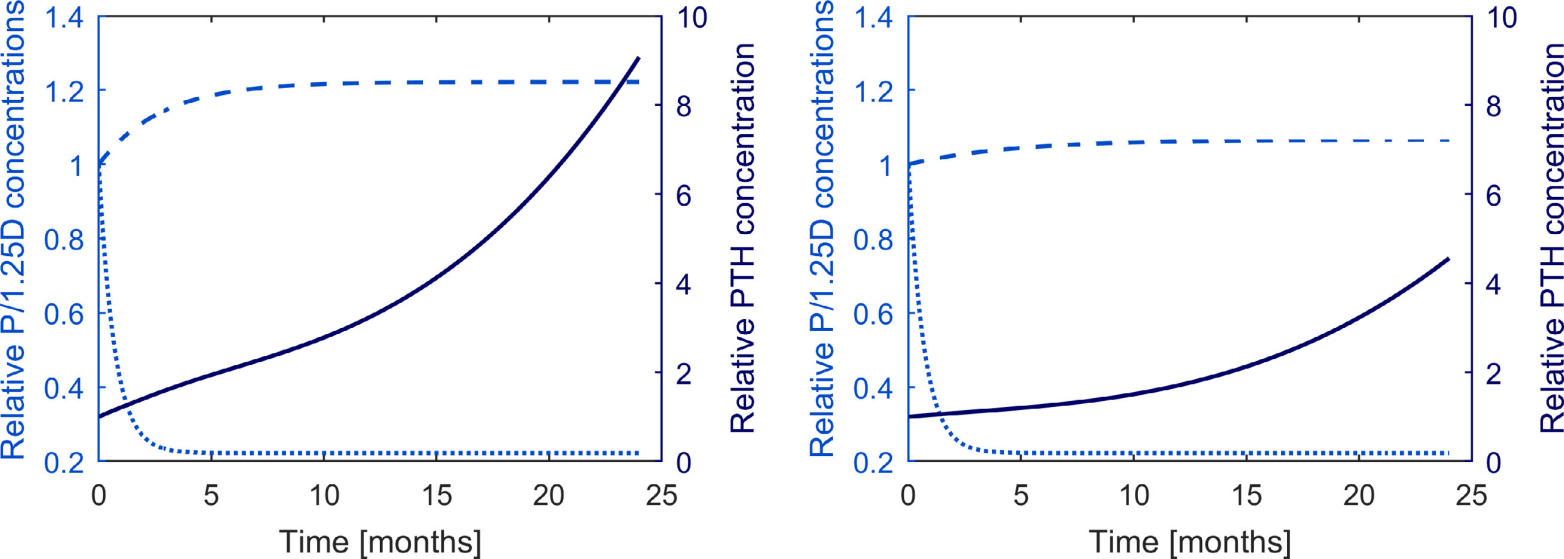
Time versus phosphate (dashed line) and 1,25D concentrations (dotted line) and the corresponding PTH concentrations (solid line) simulating experimental setup reported in Rutherford et al. (1977). High phosphate levels (left panel) lead to significantly higher PTH values than moderate elevated phosphate levels (right panel).

A steady increase of PTH can also be observed if Ca^2+^ is kept at an optimal value, but 1,25D is reduced (e.g., due to impaired synthesis), and phosphate levels are elevated (e.g., due to impaired clearance). Although there is no prominent PTH peak because Ca^2+^ is maintained at the optimal level, the PTH concentration will continuously increase and not reach a steady state due to the loss of CaSR expression and sensitivity and the increased cellular proliferation. In 1977, Rutherford and colleagues (Rutherford et al. 1977) reported long‐term effects of a regular phosphate diet and a low phosphate diet on PTH levels in 5/6 nephrectomized dogs. PTH levels of dogs on regular phosphate intake rose steadily over the 2 years; the endpoint being almost 10‐fold above the normal levels. An increase in the slope was significantly lower if phosphate intake was lowered in line with the decreased glomerular filtration rate (Fig.**** 12). We predict the same effect with our model. We observe a slower incline if phosphate is elevated only slightly over time (Fig. 13).

## Discussion

Predictions generated with our model agree with observed PTH response and PTG adaptation in a number of acute and chronic alterations of Ca^2+^, P, and 1,25D. The model accurately accounts for adaptation mechanisms such as decreased intracellular PTH degradation, PTH production rate, and elevated PTG proliferation rate. More importantly, it fully captures the different timescales, these mechanisms are acting on. The driving force of the PTG model is the description of changes in the CaSR which is the crucial regulator of the PTG. We validated our model by comparing its predictions with published experimental data under different conditions. The model predictions agree with observations in all analyzed scenarios, that is, different rates of induced hypocalcemia (Brent et al. 1988), hysteresis (Conlin et al. 1989; Schwarz et al. 1998), hyperplasia in the case of induced chronic mild hypocalcemia (Brown 2015), and secondary hyperparathyroidism in the case of chronically elevated serum phosphate levels as well as chronically low 1,25DH levels (Fukagawa et al. 1990; Quarles et al. 1994; Slatopolsky et al. 1999; Tokumoto et al. 2005; Brown 2015). This feature makes the model particularly interesting in patients suffering from secondary hyperparathyroidism, such as those with CKD or on dialysis.

To our best knowledge, the proposed model is the first to include all of the known adaptive mechanisms which regulate the CaSR. The predictions are based on positive and negative feedback systems acting on the CaSR. The effect of therapeutic interventions acting on the CaSR, such as the calcimimetic drugs cinacalcet or etelcalcetide (Block et al. 2004; Valle et al. 2008; Padhi and Harris 2009), can be easily incorporated by using an operational model of allosterism on the CaSR (Leach et al. 2007, 2010, 2016; Roche et al. 2013). Therefore, the model provides a complementary tool to study treatment strategies like combinations of calcimimetics and vitamin D analogs (Teng et al. 2003).

A limitation of the model is the control of extracellular levels of ionized Ca^2+^, 1,25D, and phosphate since in reality these concentrations are themselves affected by PTH release. We deliberately accept this limitation for the sake of clarity. The control of the parameters provides us the opportunity to study isolated effects of alterations in each individual parameter on the parathyroid gland biology. However, since these key regulators of PTG cells in hemodialysis patients are the only input parameters, the model can be easily combined with a bone model, for example (Peterson and Riggs 2010), allowing the analysis of a highly complex system involving a cascade of regulatory triggers and feedback loops.

Another regulator of phosphate and calcium homeostasis, as well as PTH release in healthy subjects, is fibroblast growth factor 23 (FGF23) (White et al. 2000; Shimada et al. 2001). FGF23 decreases renal phosphate reabsorption and thus increases renal phosphate excretion. Due to a negative feedback loop with 1,25D, FGF‐23 regulates intestinal phosphate and calcium absorption (Shimada et al. 2004; Liu et al. 2006; Quarles 2012). Moreover, FGF23 inhibits PTH secretion and synthesis (Ben‐Dov et al. 2007), whereas PTH stimulates FGF23 creating another negative feedback loop (Kawata et al. 2007). At early stages of CKD, FGF23 prevents hyperphosphatemia by promoting phosphaturia and decreasing 1,25D leading to a reduction in phosphate absorption and enhancement of renal phosphate excretion (Gutierrez et al. 2005; Jueppner et al. 2010). However, as renal function declines, the increased FGF23 can no longer increase phosphate excretion. The role of FGF23 in dialysis patients, with negable phosphate excretion, is not clear. While FGF23 is associated with poor clinical outcome (Isakova et al. 2011), it might not be an independent predictor (Isakova et al. 2015). Despite up to 100‐fold elevated levels of FGF23 in hemodialysis patients, most of these patients develop severe secondary hyperparathyroidism (Komaba and Fukagawa 2012). In a recent study, Mace and coworkers (Mace et al. 2018) showed that FGF23 inhibits PTH synthesis and secretion in normocalcemia. However, during acute hypocalcemia, where increased PTH levels are necessary to reverse hypocalcemia, no inhibitory effect of FGF23 on PTH secretion was observed. Hence, despite having a crucial role for phosphate homeostasis in healthy subjects, due to the absence of a functioning kidney and the failing downregulation of PTH, secretion of FGF23 is omitted in this model.

While we modeled the situation of hemodialysis patients with total loss of the kidney function as a system regulator, the model is more generally applicable to other patients groups, for example, patients suffering from primary hyperparathyroidism. The only parameter which is directly influenced by the kidney function is the clearance rate, *k*_cl_. Since PTH is cleared by the liver and the kidney, the clearance rate is higher in healthy subjects (Table 1) or CKD patients with residual kidney function. For our long‐term simulations, we assumed that the clearance rate is reduced by a factor 2. However, in the well‐controlled system present in healthy subjects, the model is significantly simplified in that calcium homeostasis is quickly restored in the presence of disturbances. As a result, the long‐term adaptation mechanisms are of no importance.

In this model, we capture the physiological regulation of the parathyroid gland. Further efforts will be undertaken to include pharmacological control of the PTG such as the effect of calcimimetics on PTH levels. The pharmacological control of PTG involves some adjustments such as the reversibility of hyperplasia due to the administration of calcimimetics (Drueke et al. 2007; Jean et al. 2007; Meola et al. 2009).

In conclusion, our model captures all the major regulators of PTG biology which are operative in patients with CKD and those on hemodialysis, thereby allowing us to study a multitude of sequelae, including some aspects of secondary hyperparathyroidism. Furthermore, the model can be extended with mathematical models describing bone remodeling and intestinal calcium and phosphate absorption, thus allowing a detailed study of CKD‐MBD.

## Conflict of Interest

Peter Kotanko holds stock in Fresenius Medical Care.
